# Capsaicin Promotes Apoptosis and Inhibits Cell Migration via the Tumor Necrosis Factor-Alpha (TNFα) and Nuclear Factor Kappa B (NFκB) Signaling Pathway in Oral Cancer Cells

**DOI:** 10.7759/cureus.69839

**Published:** 2024-09-21

**Authors:** Niranjana Arivalagan, Abinaya Ramakrishnan, Jospin Sindya, Jeevitha Rajanathadurai, Elumalai Perumal

**Affiliations:** 1 Medicine, Saveetha Medical College and Hospitals, Saveetha Institute of Medical and Technical Sciences, Saveetha University, Chennai, IND; 2 Ophthalmology, Saveetha Medical College and Hospitals, Saveetha Institute of Medical and Technical Sciences, Saveetha University, Chennai, IND; 3 Center for Global Health Research, Saveetha Medical College and Hospitals, Saveetha Institute of Medical and Technical Sciences, Saveetha University, Chennai, IND

**Keywords:** anti-cancer drug discovery, capsaicin, kb1 cell line, mtt assay, oral cancers, apoptosis

## Abstract

Background

Oral squamous cell carcinoma (OSCC) is a highly prevalent cancer worldwide. Microbial infections, poor oral hygiene, and chronic viral infections such as human papillomavirus (HPV) contribute to its incidence. Capsaicin, known for its presence in chili peppers, has demonstrated potential antiproliferative effects in cancer cells. It operates by inducing programmed cell death, regulating the expression of transcription factors, halting cell cycle progression, and influencing growth signal transduction pathways. These findings suggest capsaicin's promising role as a candidate for further exploration in combating oral cancer.

Aim

This study intends to identify and evaluate the anticancer properties of capsaicin on oral cancer cells through in vitro investigations.

Methodology

Using the 3-(4,5-dimethylthiazol-2-yl)-2,5-diphenyltetrazolium bromide (MTT) technique, the cell viability of oral cancer cells treated with capsaicin was evaluated. Capsaicin was applied to the KB1 cells in a range of concentrations (25-150 µg/mL) over 24 hours. The morphological alterations of the cells were assessed using a phase contrast microscope. Nuclear factor kappa B (NFκB) and tumor necrosis factor-alpha (TNFα) were subjected to quantitative real-time polymerase chain reaction (PCR) gene expression analysis. To investigate nuclear morphological changes, oral cancer cells were stained with acridine orange/ethidium bromide (AO/EtBr). The apoptotic nuclei were visualized using a fluorescent microscope. A scratch wound healing test was performed to check for capsaicin’s anti-migratory potential.

Result

In our investigation of oral cancer cells treated with capsaicin, there was a significant drop in cell viability between the control and treatment groups (p < 0.05). The inhibitory concentration (IC50) was found to be 74.4 μM/mL in oral cancer cells. Following treatment, fewer cells were present, and those that were present shriveled and exhibited cytoplasmic membrane blebbing. Under AO/EtBr staining, treated cells exhibited chromatin condensation and nuclear disintegration. Furthermore, the migration of capsaicin-treated cells was significantly lower than that of control cells. The results of gene expression analysis demonstrated a considerable downregulation of TNFα and NFκB following capsaicin administration.

Conclusion

The study's findings suggest that capsaicin may have anti-tumor properties in oral cancer cells. More research is desperately needed to fully understand the mechanism underlying capsaicin's anticancer potential and therapeutic applicability.

## Introduction

Globally, the most prevalent cancer is oral squamous cell carcinoma (OSCC). It is important to note that 25% of people who develop oral cancer don’t smoke or have other known risk factors. It is the most prevalent type of head and neck cancer [1]. It typically affects adults aged 60 and above. Oral cancer affects the lips, the tip of the tongue, the roof of the mouth, and the floor. It also affects the oropharynx, tonsils, and the sides and rear of the throat [2]. Oral cancer makes up 90% of all oral cavity cancers. It predominates in all cases with potentially malignant conditions such as inflammatory oral submucosa, fibrosis, erythroplakia, leukoplakia, candidal leukoplakia, and lichen planus, all of which are markers of the preclinical stage of oral cancer [3].

Asia has the highest incidence of oral cancer. In India, estimates for lip and oral cavity cancer in 2022 were 79,979 deaths and over 143,759 cases. Globally, 377,713 new cases and 177,757 deaths were estimated in 2020 [4]. The biggest issue for community health is the rising number of occurrences of oral cancer. The incidence of oral cancer in India is much greater than in other countries, as approximately 70% of cases are typically reported in later stages, such as Stage III-IV, where there is only a five-year survival rate due to the extremely low possibility of a cancer cure. Physical examination includes an examination of the entire mouth and an examination of the head, face, and neck for any signs of pre-cancer or malignancy. A brush biopsy (also known as scrape biopsy or exfoliative cytology) can be performed to acquire cells to be checked for malignancy by gently scraping the affected area using a small brush or spatula. Furthermore, an incisional biopsy can be utilized to remove small bits of tissue to obtain cells for cancer testing. Indirect laryngoscopy and pharyngoscopy can also be performed to examine the throat, the base of the tongue, and a portion of the larynx (voice box).

Chemotherapy, surgery, and radiation therapy are among the options for treating oral cancer today. However, these methods have their limitations. The challenges in administering chemotherapy include the possibility of toxicity to the remaining healthy cells. Despite the fact that patient survival rates have increased with the use of modern chemotherapeutic drugs, treatment outcomes are still subpar because of the emergence of serious side effects and drug resistance [5]. There are plant-based drugs used for the treatment of oral cancer, such as *Azadirachta indica* and *Senegalia catechu*. The cytotoxic and anticancer effects of *S. catechu* have been reported in COLO-205, HT-1080, and HeLa cell lines in vitro. *Dracaena cinnabari*, in a recent in vitro study, was analyzed for its apoptosis-inducing and cytotoxic effects against the OSCC (H400) cell line. *Piper nigrum* L. presents cytotoxic activity against the human oral squamous carcinoma (HOSC) KB cell line [5-8].

Capsaicin (trans-8-methyl-N-vanillyl-6-nonenamide) is the main pungent component of the fruits of capsicum plants in the nightshade family, *Solanaceae*. Capsaicins are found in chili peppers, and the main active compound is capsaicin. Numerous research investigations have documented capsaicin's antiproliferative effects on cancer cells, primarily through the activation of programmed cell death, modulation of transcription factor expression, and halting cell cycle progression. Because it can promote cell death and mediate cell cycle arrest, capsaicin has been demonstrated to be cytotoxic in a variety of cancer cell types, including hepatocarcinoma, breast cancer, colon cancer, and non-small cell lung cancer. Moreover, in lipopolysaccharide (LPS)-stimulated RAW 264.7 cells, capsaicin inhibits the production of the pro-inflammatory cytokine tumor necrosis factor-alpha (TNFα), and this inhibitory effect is linked to peroxisome proliferator-activated receptor gamma (PPARγ) activation. Capsaicin may be a naturally occurring PPARγ ligand that could help treat inflammatory illnesses [9].

Capsaicin has been extensively studied in relation to various cancers, yet its impact on oral cancer remains relatively unexplored. This study seeks to assess the anticancer potential of capsaicin specifically on oral cancer cells in vitro.

## Materials and methods

Cell line maintenance

The normal fibroblast (3T3) and oral cancer cell lines (KB1) were obtained from the National Centre for Cell Science (NCCS), Pune. Cells were grown in T25 culture flasks using Dulbecco's Modified Eagle Medium (DMEM) supplemented with 10% fetal bovine serum (FBS) and 1% antibiotics (penicillin-streptomycin). To maintain the cells at 37°C, a humidified environment with 5% CO₂ was used. Once 80% confluence was achieved, the cells were trypsinized and passaged.

Cell viability assay

The 3-(4,5-dimethylthiazol-2-yl)-2,5-diphenyltetrazolium bromide (MTT) test was used to assess the viability of oral cancer cells following capsaicin therapy. The approach is based on the notion that metabolically active cells convert soluble yellow tetrazolium salt into insoluble purple formazan crystals. Plating was done in 96-well plates with 5 x 10^3^ cells per well. Twenty-four hours after plating, cells were washed twice with 100 μL serum-free media and starved for three hours at 37°C. Following starvation, cells were treated with various concentrations of capsaicin (25-150 μM/mL). The medium from the control and capsaicin-treated cells was withdrawn after 24 hours of treatment, and 100 μL of DMEM containing MTT (0.5 mg/mL) was added to each well. For four hours, cells were maintained in a CO_2_ incubator at 37°C. After removing the MTT-containing media, the cells were washed with 1X phosphate-buffered saline (PBS; pH: 7.2). Following a one-hour incubation period in the dark, 100 μL of dimethyl sulfoxide (DMSO) was used to dissolve the formazan crystals. Next, a micro-enzyme-linked immunosorbent assay (ELISA) plate reader set to 570 nm was used to measure the intensity of the color. As a proportion of control cells grown in media without serum, the number of viable cells was expressed. In the control media, there was no treatment, and cell viability was 100%. The formula for calculating cell viability is:

\[ \text{Cell viability (%)} = \frac{\text{A}_{570 \, \text{nm}} \text{ of treated cells}}{\text{A}_{570 \, \text{nm}} \text{ of control cells}} \times 100 \]

Morphology analysis

For morphology analysis, 2 × 10^5^ cells seeded in six-well plates were treated with capsaicin at the optimal dosage obtained from MTT assay (inhibitory concentration, IC50: 74.4 µM/mL) for 24 hours. The media was removed from the cells during the incubation period, and they were washed once with PBS (pH 7.4). The plates were inspected with a phase contrast microscope.

Staining

Capsaicin's effects on oral cancer cell death were investigated using the acridine orange/ethidium bromide (AO/EtBr) dual staining approach. After a 24-hour capsaicin treatment, the cells were collected and washed in ice-cold PBS. The pellets were suspended in 1 mg/mL AO and 1 mg/mL EtBr (5 µL each). A fluorescent microscope was then utilized to observe the apoptotic changes in the stained cells.

Scratch wound healing test

Oral cancer cells (2 × 10^5^ per well) were seeded in six-well culture plates. Under an inverted microscope, a 200 μL tip was used to make an incision in the cell monolayer, followed by a wash with PBS. After administering the IC50 dose and serum-free culture media to the treatment and control cells, respectively, for 24 hours, the damaged area was imaged under the same microscope. In addition, this step was repeated three times.

Real-time polymerase chain reaction (PCR)

The expressions of the TNFα and NFκB genes were investigated by real-time PCR. The conventional technique of using Trizol Reagent (Sigma-Aldrich, St. Louis, MO, USA) was utilized to extract total RNA. A 2 μg sample of RNA was reverse-transcribed to produce cDNA using the PrimeScript First Strand cDNA Synthesis Kit (TakaRa, Shiga, Japan). Specific primers were employed to amplify the targeted genes. The PCR was carried out using the GoTaq® qPCR Master Mix (Promega Corporation, Madison, WI, USA), which includes SYBR Green dye in addition to other PCR ingredients. A Bio-Rad CFX96 PCR (Bio-Rad Laboratories, Hercules, CA, USA) system was used to conduct RT-PCR. The comparative CT approach was employed to evaluate the data, and the Schmittgen and Livak 2−∆∆CT method was utilized to calculate the fold change.

Statistical analysis

All data obtained were analyzed by one-way analysis of variance (ANOVA) followed by Student’s t-test using PRISM software version 4 (GraphPad Software, Inc., San Diego, CA, USA), represented as mean ± standard deviation (SD) for triplicates. The level of statistical significance was set at a p-value < 0.05.

## Results

Cytotoxic effects of capsaicin on 3T3 fibroblasts and pro-apoptotic potential on KB1 oral cancer cells

**Figure 1:**
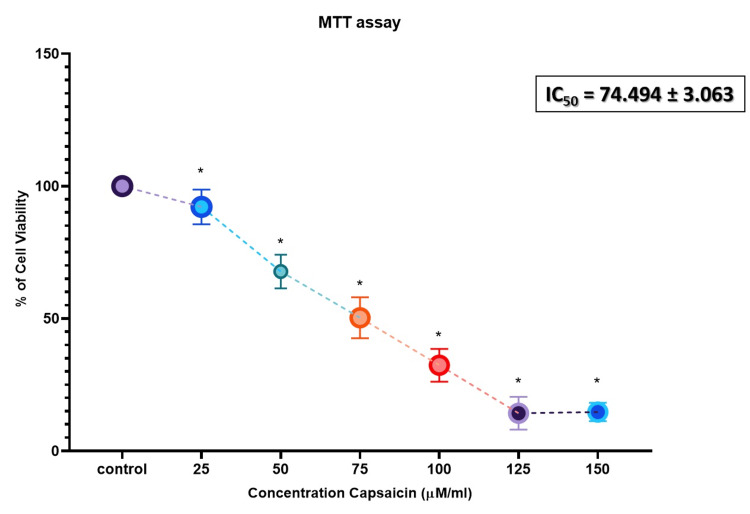
Capsaicin’s pro-apoptotic effect in oral cancer cell line The MTT test was used to measure cell viability after 24 hours of treatment with capsaicin (25-150 μM/mL). The data is given as mean ± SD (n = 3). * A p-value of <0.05 indicates the statistical significance between the treatment group and the control group. MTT: 3-(4,5-dimethylthiazol-2-yl)-2,5-diphenyltetrazolium bromide; IC50: 50% inhibitory concentration; SD: Standard deviation

**Figure 2:**
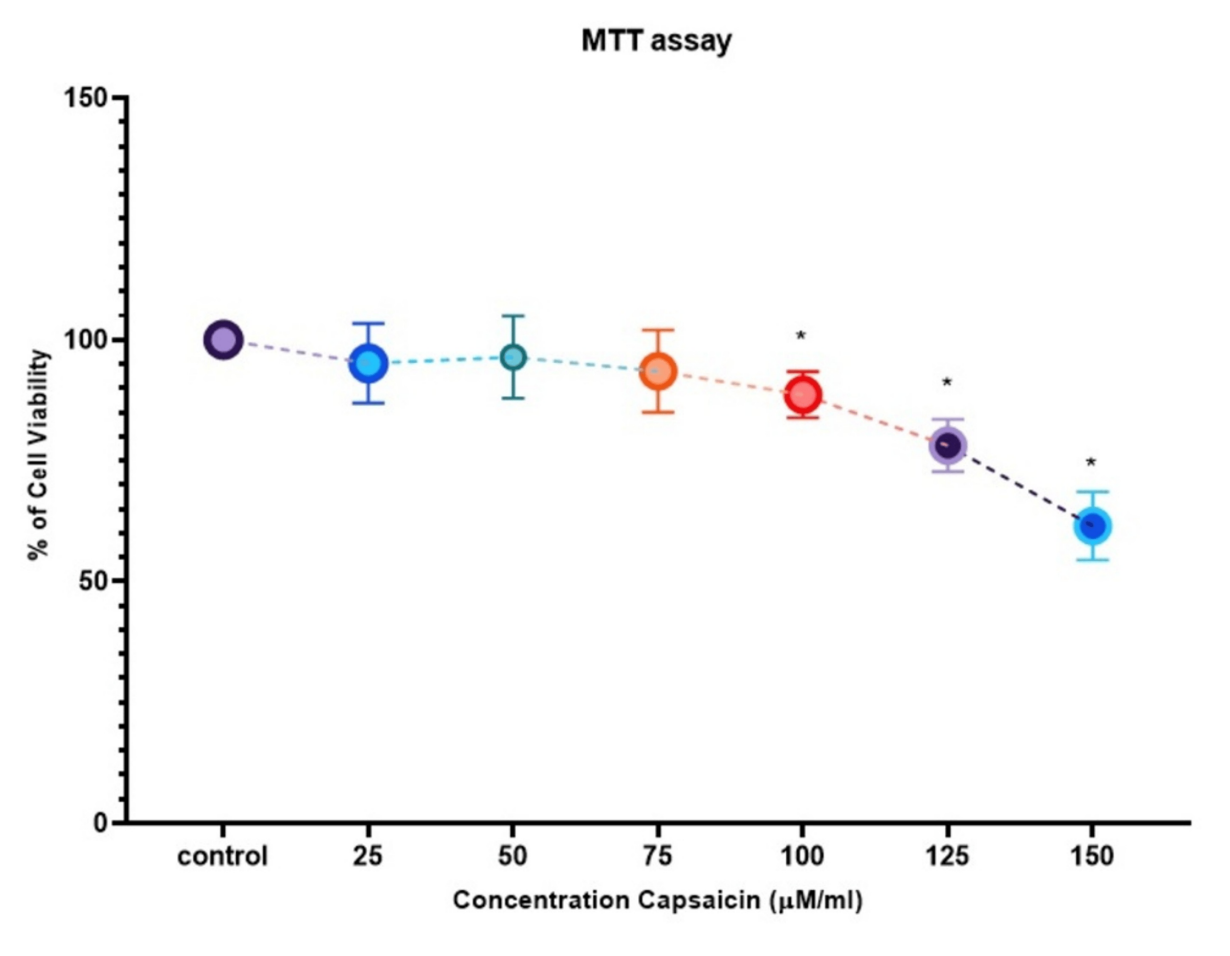
Cytotoxic effect of capsaicin in normal fibroblast cell line MTT test was used to measure cell viability after 24 hours of treatment with capsaicin (25-150 μM/mL). The data is given as mean ± SD (n = 3). * A p-value of <0.05 indicates the statistical significance between the treatment group and the control group. MTT: 3-(4,5-dimethylthiazol-2-yl)-2,5-diphenyltetrazolium bromide; IC50: 50% inhibitory concentration; SD: Standard deviation

The cells were exposed to various doses of capsaicin, ranging from 25 to 150 µM/mL, for 24 hours. Our study found that capsaicin significantly decreased the viability of KB1 oral cancer cells compared to the control group at this time point. As the concentration increased, the number of viable cells progressively declined. The IC-50​ value, representing a 50% growth inhibition, was determined to be 74.4 µM/mL and was used for further investigations (Figure 1). In contrast, normal fibroblasts did not exhibit a significant reduction in viability following capsaicin treatment (Figure 2).

Morphological observations

**Figure 3:**
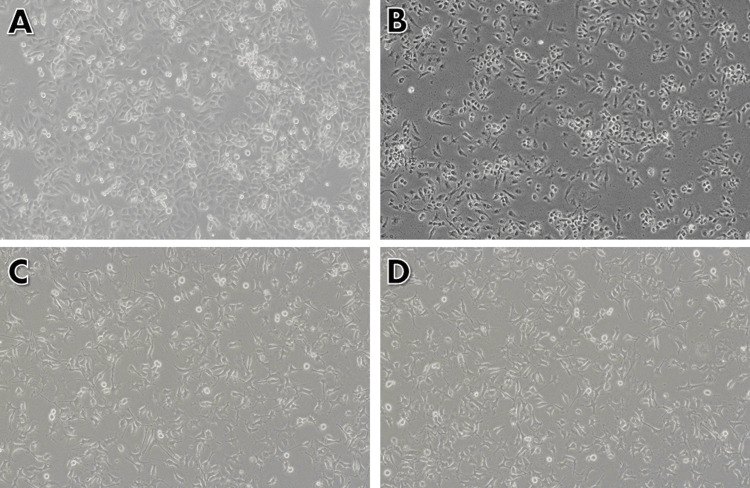
The effect of capsaicin on the morphology of human oral cancer cells (KB1) and normal fibroblast cells (3T3) After being exposed to capsaicin (74.4 μM/mL) for 24 hours, the cells were examined using an inverted phase-contrast microscope. (A) Control cells (KB1) without capsaicin treatment; (B) KB1 cells after 24 hours of treatment with capsaicin (74.4 μM/mL) showing cell shrinkage and cytoplasmic membrane blebbing; (C) Control cells (3T3) without capsaicin treatment; (D) 3T3 cells after 24 hours of treatment with capsaicin (74.4 μM/mL)

After 24-hour exposure to 74.4 µM/mL of capsaicin, the KB1 oral cancer cell line exhibited significant morphological changes compared to untreated cells. These changes included cell atrophy and a reduced cell count, indicative of apoptosis. Dying cells displayed further alterations, such as becoming rounder, contracting, and losing contact with adjacent cells. Additionally, some of the fragile cells detached from the plate surface. In contrast, no morphological changes were observed in 3T3 cells (Figure 3).

Identification of apoptotic cells

**Figure 4:**
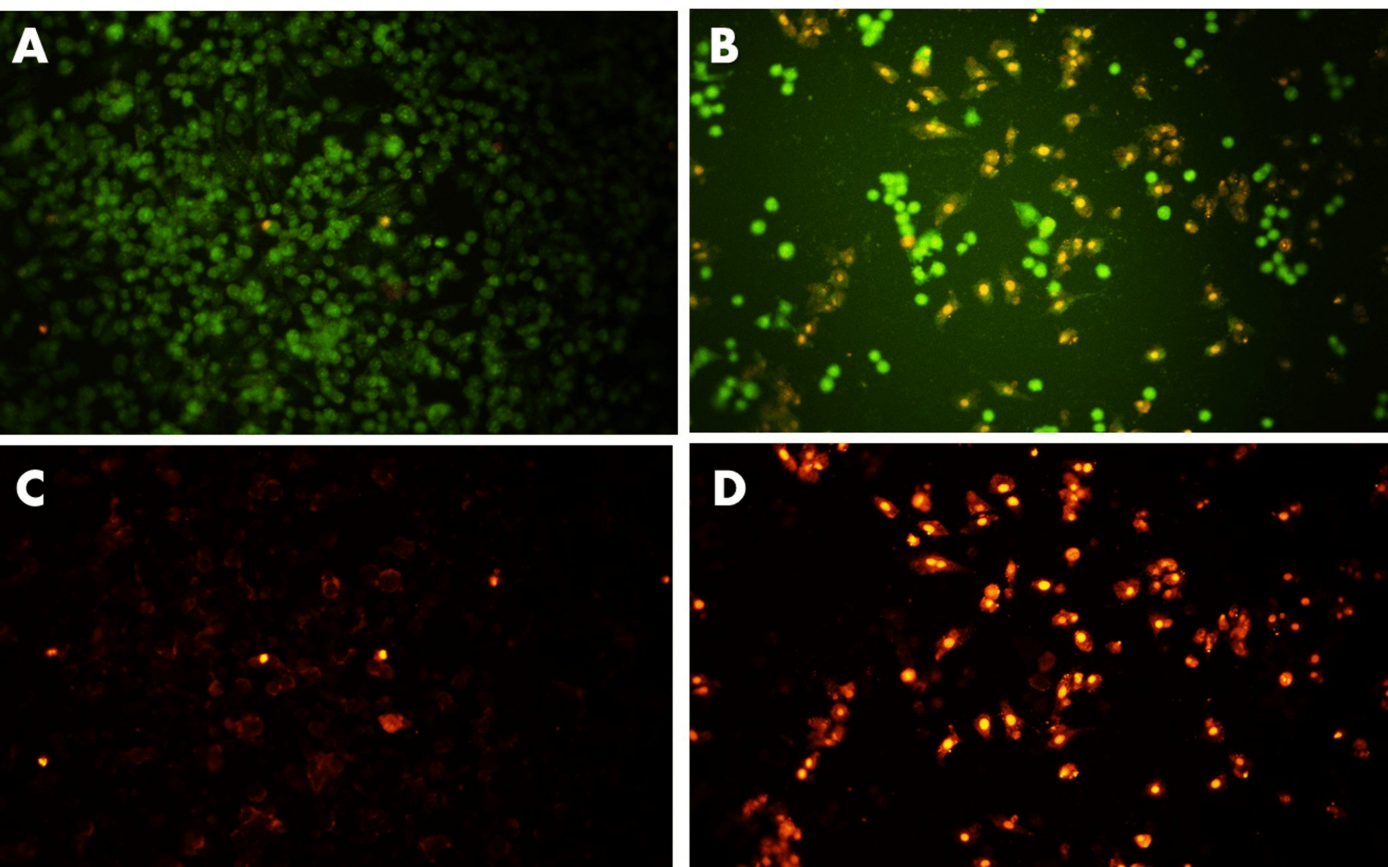
Detection of apoptotic cells in capsaicin (74.4 µM/mL)-treated oral cancer cells by AO/EtBr dual staining Oral cancer cells were treated with capsaicin (74.4 µM/mL) for 24 hours. Following treatment, the cells were subjected to dual staining with AO and EtBr. An inverted fluorescent microscope was used to obtain the images. Control cells displayed a consistent green hue and the cells treated with capsaicin extract displayed yellow, orange, and red signals. (A) AO-stained control cells without capsaicin treatment; (B) AO-stained cells after capsaicin treatment for 24 hours; (C) EtBr-stained control cells without capsaicin treatment; (D) EtBr-stained cells after capsaicin treatment for 24 hours AO: Acridine orange; EtBr: Ethidium bromide

After 24 hours of exposure to capsaicin (74.4 µM/mL), the nuclear appearance of apoptotic cells was investigated using AO/EtBr dual staining. After 20 minutes of staining with AO/EtBr dye, the treated cells were examined under fluorescence microscopy. The observed results demonstrated that while AO tagged both living and dying cells, both equally showed green fluorescence, whereas EtBr only marked cells that had lost their membrane integrity. Cells dyed yellow or orange indicate that they are in an apoptotic state, while cells labeled green indicate that they are still alive. In the current study, cells treated with capsaicin extract showed signals in yellow, orange, and red colors, while control cells consistently showed a green tint (Figure 4). These results show that in KB1 oral cancer cells, capsaicin extract induces apoptosis.

Scratch wound healing assay

**Figure 5:**
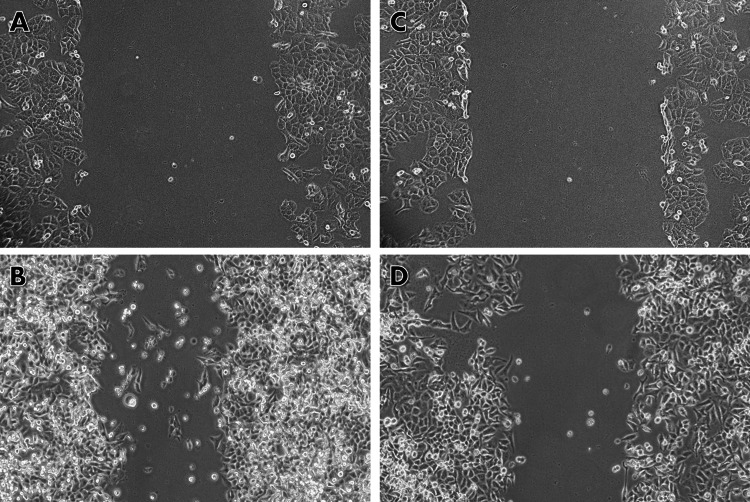
Scratch wound healing test An in vitro scratch wound healing experiment was utilized to assess capsaicin's anti-migratory properties. After damaging oral cancer (KB1) cells, a 24-hour cell migration experiment was performed with and without capsaicin (74.4 µM/mL) treatment. The images were obtained using an inverted phase-contrast microscope. (A) Cells without capsaicin treatment at zero hours after scratching; (B) Deliberately migrated cells without capsaicin treatment, 24 hours after scratching; (C) Capsaicin (74.4 μM/mL)-treated cells at zero hours after scratching; (D) Capsaicin (74.4 μM/mL)-treated cells at 24 hours after scratch introduction showing restricted migration

Cell migration is a characteristic feature of cancer metastasis and is required for tumor invasion and dissemination. Cancer cells can migrate by altering their cytoskeletal dynamics and adhesion properties, allowing them to separate from the initial tumor and invade neighboring organs. To identify potential therapeutic targets and create novel anti-cancer therapies, studies on metastasis and cell movement must be conducted in a controlled cell culture environment. A scratch test was used to determine how capsaicin affected lung cancer cell migration. The results showed that capsaicin suppresses cell migration when compared to control cells. The following observations were made: after 24 hours, untreated cancer cells in the control group had spread to cover the scratched region. Capsaicin (74.4 μM/mL) significantly decreased lung cancer cell migration compared to the control group. The cell migration distance was shorter in the capsaicin-treated group compared to the control group (Figure 5).

Gene expression of TNFα and NFκB

**Figure 6:**
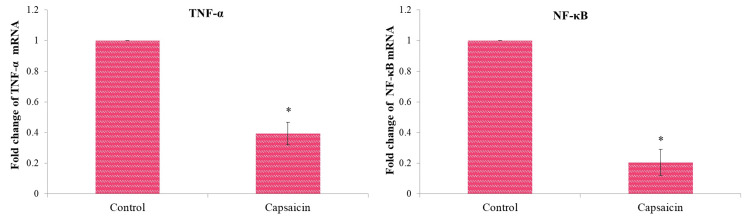
Effect of capsaicin (74.4 μM/mL) on the gene expression of (A) TNFα and (B) NFκB in oral cancer cell After normalizing target gene expression to GAPDH mRNA expression, the results are presented as a fold change from control. Each bar represents the mean ± standard deviation of three different observations. * A p-value of <0.05 indicates statistical significance between the drug-treated and control groups. TNFα: Tumor necrosis factor alpha; NFκB: Nuclear factor kappa B; GAPDH: Glyceraldehyde 3-phosphate dehydrogenase

TNFα and NFκB genes are considered markers of KB1 oral cancer cells. Anti-apoptosis is thought to play an important role in cancer cell proliferation and metastasis. Capsaicin treatment significantly reduced the expression of the anti-apoptotic genes TNFα and NFκB in oral cancer cells (Figure 6). The inhibition of oral cancer cell migration was connected to the downregulation of these anti-apoptotic genes. 

## Discussion

Many studies have been conducted in the past few years to create novel chemotherapy medications. The creation of new compounds for therapeutic application has not greatly increased despite efforts in drug discovery. Globally, oral cancer is the most common type of cancer. Numerous plant-based medications have been proven to be successful in treating cancer, including naringin [10], propolis [11], noscapine [12], curcumin [13], and quercetin [14]. This study clarifies the possible therapeutic uses of capsaicin in the treatment of oral cancer by examining the intricate molecular pathways underlying the compound's diverse effects on oral cancer cells. These findings provide valuable insights into the apoptotic pathways that capsaicin impacts, advancing our understanding of the compound's anti-cancer properties.

The KB1 oral cancer cell line and the 3T3 normal fibroblast cell line were utilized in this work to examine the cytotoxic and pro-apoptotic properties of capsaicin. For 24 hours, the cell lines were exposed to varying concentrations of capsaicin (25-150 μM/mL) in order to evaluate its growth-inhibitory effects. Based on dosage and time, our data demonstrated a significant reduction in KB1 cell viability with capsaicin therapy. Higher dosages of capsaicin (74.4 µM/mL) resulted in significant cell death, indicating potential pro-apoptotic activity. Utilizing phase-contrast microscopy, the anticancer potential was verified.

Apoptosis, a synonym for programmed cell death, is defined by DNA breakage, chromatin condensation, cell shrinkage, and the activation of certain enzymes called caspases [15]. By obstructing the apoptotic process, cancer spreads. Inducing apoptosis in tumor cells is the most efficacious therapeutic method and is employed in numerous cancer treatments. Since capsaicin significantly raised the proportion of apoptotic cells, our results point to its pro-apoptotic role.

Numerous studies have shown that capsaicin is beneficial against several forms of cancer, such as lung cancer [16,17], colorectal cancer [18], prostate cancer [19], bladder cancer [20], carcinoma of the nose [21], and cholangiocarcinoma [22]. Our results show that capsaicin therapy increases the proportion of apoptotic cells considerably, exhibiting a pro-apoptotic effect. The existence of capsaicin-induced apoptotic cells in oral cancer cell lines was confirmed by AO/EtBr staining.

Limitations

There are limitations to in vitro studies of capsaicin’s anticancer effects. These studies often fail to accurately replicate the complexities of the human body, including organ interactions and immune responses. Consequently, promising in vitro results may not always translate to effectiveness or safety in living organisms. Further research, including in vivo studies and investigations into the molecular mechanisms of capsaicin’s action, is necessary to better understand its therapeutic potential and bridge the gap between laboratory findings and clinical applications.

## Conclusions

This study investigated the effects of capsaicin on oral cancer cell lines, with a particular emphasis on the TNFα and NFκB signaling pathways. The in vitro analysis revealed capsaicin's anticancer and anti-migratory properties, demonstrating its influence on key apoptotic pathways by modulating the gene expression of TNFα and NFκB. These results provide valuable insights into capsaicin's potential clinical applications for treating oral cancer and contribute to the expanding research on natural compounds as possible adjuncts in cancer therapy. The study highlights capsaicin's potential as a cytotoxic and pro-apoptotic phytochemical that affects TNFα and NFκB gene expression, underscoring its promise as a therapeutic agent for oral cancer. As interest in phytochemicals for cancer treatment grows, further preclinical and clinical research could position capsaicin as a significant component in oral cancer therapy.
